# Progressive Multifocal Leukoencephalopathy Initially Suspected As Brain Relapse From Classical Hodgkin’s Lymphoma

**DOI:** 10.7759/cureus.44000

**Published:** 2023-08-23

**Authors:** Akio Onishi, Ayako Muramatsu, Yuji Shimura, Taichi Murao, Takahiro Fujino, Shinsuke Mizutani, Taku Tsukamoto, Yukiko Shishido-Hara, Junya Kuroda

**Affiliations:** 1 Division of Hematology and Oncology, Department of Medicine, Kyoto Prefectural University of Medicine, Kyoto, JPN; 2 Department of Pathology and Applied Neurobiology, Kyoto Prefectural University of Medicine, Kyoto, JPN

**Keywords:** mirtazapine, mefloquine, john cunningham virus, methotrexate, progressive multifocal leukoencephalopathy, classical hodgkin lymphoma

## Abstract

HIV-negative progressive multifocal leukoencephalopathy (PML) has a poor prognosis due to a lack of standard treatment. Herein, we report a patient with HIV-negative PML which occurred after the treatment for classical Hodgkin’s lymphoma (CHL). A 71-year-old male patient was admitted to our hospital due to various neurological symptoms, including memory disturbance, dysgraphia, ataxia, and ideomotor apraxia, at 16 months after high-dose salvage chemotherapy with autologous peripheral blood stem cell transplantation (PBSCT) for primary treatment-refractory CHL. The patient’s blood and serological examination results were mainly normal, including CD4-positive T lymphocyte count and serum immunoglobulin levels. T2-weighted fluid-attenuated inversion recovery MRI showed high-intensity lesions from the left occipital lobe to the corpus callosum. Moreover, the rapid intraoperative pathological assessment of biopsy specimens obtained from abnormal brain lesions suggested brain relapse of CHL. The patient’s symptoms progressed rapidly; therefore, treatment with high-dose methotrexate was started, which significantly improved the patient’s symptoms and MRI findings within a week. However, further examinations of the biopsy specimens with in situ hybridization and immunohistochemical examinations showed reactivation of the John Cunningham virus (JCV) in the astrocytes. Further, cells initially believed to be Hodgkin cells based on the rapid intraoperative pathological assessment were found to be destructive astrocytes, thereby confirming the diagnosis of PML. The patient was then successfully treated with combined mefloquine and mirtazapine and did not have any fatal outcomes. Based on this case, a differential diagnosis of PML from CNS involvement of CHL is important even in cases without evident biomarkers for immunodeficiency. Moreover, methotrexate was likely to be effective in improving neurological symptoms by decreasing brain parenchyma inflammation in the acute phase in this particular patient.

## Introduction

Progressive multifocal leukoencephalopathy (PML) is an infectious disease of the central nervous system caused by the John Cunningham virus (JCV). Impaired cellular immunity causes the uncontrollable growth of JCV by reactivating subclinical JCV infection with the decline in host immunocompetence and lytic infection of oligodendroglial cells, thereby resulting in specific injury to oligodendroglial cells and the formation of demyelinating nests in the host [1,2]. In terms of clinical course, PML can lead to subacute progression. However, the prognosis is dismal in the absence of established treatment with a three-month mortality rate of 20-50% in untreated patients. In addition, the prognosis of PML may depend on the underlying disease [3].

Classically, PML occurs in individuals with immunodeficiency, most commonly those with acquired immunodeficiency syndrome with CD4-positive T lymphocyte counts of <100 cells/μL. However, recently, the emergence of PML in the non-HIV setting has been increasing. In addition, the rate of allogeneic organ transplantation and the use of biological agents for inflammatory diseases or immunosuppressive monoclonal antibodies for different conditions, including cancerous and autoimmune diseases, have increased. In approximately 10% of patients with PML in the United States of America, the underlying condition is hematological malignancy [4,5]. Further, HIV-negative PML is more common among Japanese [6,7]. In HIV-negative patients without an apparent sign or biomarker for an immunodeficient condition, PML is occasionally challenging to diagnose.

Herein, we report a patient with HIV-negative PML that emerged after treatment for classical Hodgkin’s lymphoma (CHL). The patient had a normal CD4-positive T lymphocyte count and serum immunoglobulin (Ig) level and was initially suspected of a brain relapse of CHL based on the rapid intraoperative pathologic diagnosis made according to hematoxylin and eosin staining and MRI results.

## Case presentation

**Figure 1:**
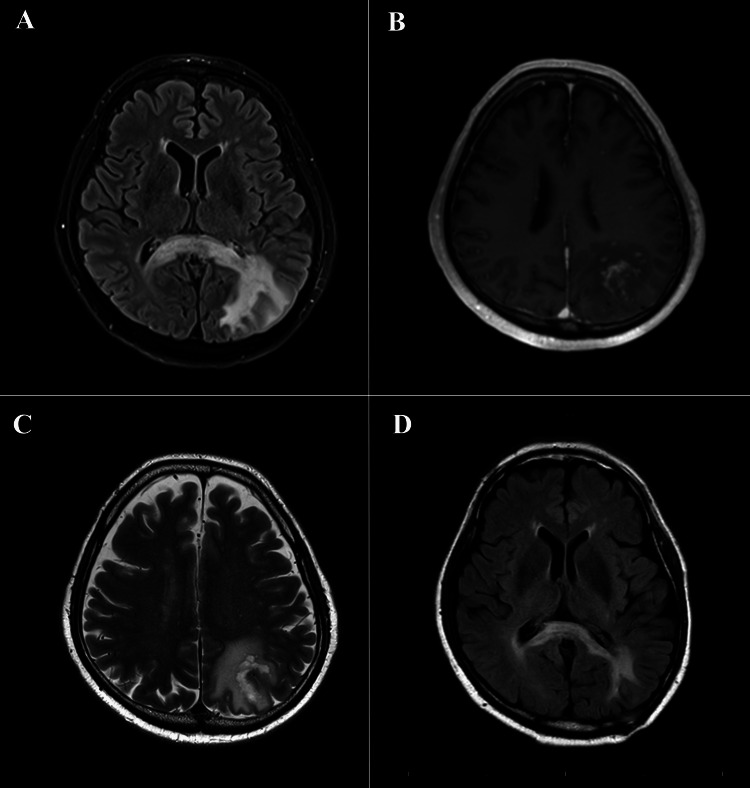
MRI findings (A) Fluid-attenuated inversion recovery, (B) gadolinium (Gd)-enhanced T1-weighted MRI, and (C) T2-weighted MRI at the onset of PML onset. (D) Gd-enhanced T1-weighted MRI after nine months of therapy with mefloquine and mirtazapine

A 72-year-old male patient was admitted to our hospital due to complaints of memory impairment, attention deficit, and lightheadedness. The patient had a history of nodular sclerosis type of CHL with the initial disease stage III according to the Ann Arbor staging system, which was primarily resistant to the first-line standard chemotherapy and required high-dose salvage chemotherapy supported by autologous peripheral blood stem cell transplantation (PBSCT) 14 months before the visit. Although the patient achieved complete remission of CHL, he experienced disseminated herpes zoster virus infection, which required treatment with acyclovir seven months after PBSCT. Moreover, he developed interstitial pneumonia caused by Epstein-Barr virus (EBV) reactivation 12 months after PBSCT. He then required treatment with prednisolone, which was administered at a starting dose of 1 mg/kg per day. At the visit, he was still treated with prednisolone at a dose of 5 mg per day. The patient also had a history of diabetes mellitus. Although his vital signs, physical examination findings, and level of consciousness were normal, he presented with dysgraphia, ataxia, and ideomotor apraxia. Nevertheless, tetraplegia and dysarthria were not observed. Peripheral blood examination showed no decrease in white blood cell count (CD3+CD4+ T lymphocyte count: 583/μL [normal range: 300-1300/μL] and CD3+CD8+ T lymphocyte count: 1,552/μL [normal range: 100-900/μL]). Serological examination revealed no pathologically significant abnormality (serum IgG level: 1153 [normal range: 861-1747] mg/dL, IgA level: 130 [normal range: 93-393] mg/dL, and IgM level: 152 [normal range: 73-109] mg/dL). The DNA tests for JCV, herpes simplex virus, or varicella-zoster virus in the peripheral blood and cerebrospinal fluid had negative results. Brain MRI showed high-signal intensity lesions from the bilateral corpus callosum to the left parietal subcortical white matter without mass effect or edema on fluid-attenuated inversion recovery (Figure 1A). In addition, a cloud-like enhancement on the gadolinium-enhanced T1-weighted image (Figure 1B) and scattered nodular lesions and a measure-like contrast effect on the T2-weighted image (Figure 1C) were observed.

**Figure 2:**
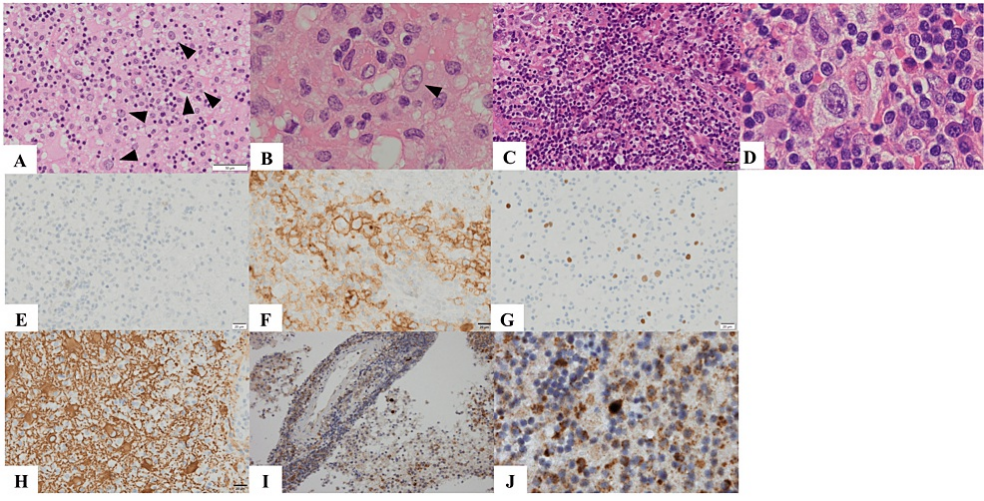
Histopathological findings (A-D) Hematoxylin-eosin staining of the brain biopsy specimens at the onset of PML (A: low magnificent view, B: high magnification view) and the lymph node biopsy specimens at the onset of CHL (C: low magnification view, D: high magnification view). Large atypical cells with enlarged nuclei (several representatives indicated by arrowheads) were scattered with a background showing different inflammatory cells (A and B). (E and F) CD30 immunohistochemical staining of the brain biopsy specimens at the onset of PML (E) and the lymph node biopsy specimens at the onset of CHL (F). (G and H) Immunohistochemical staining of OLIG2 (G) and glial fibrillary acidic protein (H) of the brain biopsy specimens. (I and J) Low (I) and high (J) magnification views of in-situ hybridization of the JCV in the brain biopsy specimen

A brain biopsy was performed, and the frozen sections of the affected brain tissues revealed the presence of atypical cells with enlarged nuclei with different inflammatory cells (Figures 2A-2B). Thus, the intraoperative pathological diagnosis was consistent with relapsed Hodgkin’s lymphoma. Indeed, the pathological findings of the brain tissues were partly similar to those of the lymph node tissues at the initial presentation (Figures 2C-2D). Because the patient’s condition rapidly deteriorated with progressive neurological characteristics, high-dose methotrexate (MTX) therapy was started before the final pathological examination, and the patient’s general and neurological conditions (constructional apraxia and disorientation) significantly improved within a week after MTX therapy. Thereafter, precise pathological assessments with immunohistochemical studies were conducted, and atypical cells, unlike Hodgkin cells, tested negative for CD30 (Figures 2E-2F) and other lymphocytic markers (data not shown). OLIG2 was positive in oligodendroglia-like cells with small nuclei. However, its immunoreactivity diminished with nuclear enlargement (Figure 2G). Immunostaining of the glial fibrillary acidic protein revealed the presence of reactive astrocytes. However, its immunoreactivity in atypical cells was unclear (Figure 2H). Intranuclear dot-shaped structures were observed in cells with enlarged nuclei, thereby indicating early signs of JCV-infected cells [8,9]. Thus, in-situ hybridization of JCV DNA was performed, and the final diagnosis of PML was determined (Figures 2I-2J).

Then, the patient was treated with mefloquine (275 mg/day for the first three days and 275 mg every other week starting on day 8 for six months) and mirtazapine [10,11]. Although the patient was free from neurological symptoms, a brain MRI revealed a high-signal area after nine months of therapy (Figure 1D). Next, 16 months after the diagnosis of PML, the patient died of systemic relapse of HL without PML re-worsening.

## Discussion

In the current case, we initially suspected the brain lesion was a post-PBSCT relapse of CHL based on the tentative pathological diagnosis via hematoxylin and eosin staining, diagnostically indefinite MRI findings, and negative cerebrospinal fluid test result for JCV. It was challenging to evaluate the immunodeficient status of a patient who presented without evident laboratory evidence of immune dysfunction. In addition, although the patient achieved a complete response with salvage high-dose chemotherapy, he was primarily refractory to conventional chemotherapy and was considered at high risk for CHL relapse. Based on these clinical characteristics, PML was not considered a differential diagnosis at the initial presentation of neurological symptoms. Nevertheless, considering his previous medical history of repeated opportunistic infections after PBSCT, caution should be taken. Several studies have reported the development of PML in CHL [12-15]. In the current case, flow cytometric analysis showed a low CD4/CD8 ratio at <1.0 in peripheral blood, thereby indicating the predominance of CD8-positive T lymphocytes, and a reactive increase in the number of plasma cells in the brain biopsy sample indicated chronic inflammatory changes. In addition, previous studies suggested the involvement of abnormal activation of CD8+ T lymphocytes in the aggravation of focal inflammation in PML [16,17]. These findings might be more indicative of PML than CHL relapse in our case.

No standard treatment strategy for PML has been established. However, previous studies have reported the efficacy of steroid pulse therapy for the neurological symptoms of PML, particularly PML-associated inflammatory responses, such as immune reconstitution inflammatory syndrome. Concerning this, it was interesting that the patient’s neurological symptoms significantly improved immediately after high-dose MTX administration. Considering the infiltration of numerous inflammatory cells in the brain lesion, MTX could have inhibited the immune reaction, thereby relieving neurological symptoms. However, there was no previous information about the efficacy of MTX for PML. Rather, continuous MTX therapy has been considered a risk of PML [18-20]. Thus, although the clinical course in our case indicated that high-dose MTX can be effective in relieving acute-phase neurological symptoms, its efficacy should be interpreted with caution. Nevertheless, this case showed the importance of controlling acute-phase neurological symptoms to achieve long-term survival.

## Conclusions

Herein, we report a rare case of non-HIV PML after PBSCT in a patient who had no evident laboratory findings indicating immunodeficiency but who presented with a medical history of opportunistic infections. The patient was successfully treated with high-dose MTX, followed by mefloquine and mirtazapine.
